# Promoter Hypomethylation of miR-124 Gene Is Associated With Major Depressive Disorder

**DOI:** 10.3389/fnmol.2021.771103

**Published:** 2021-12-21

**Authors:** Duan Zeng, Shen He, Nan Zhao, Manji Hu, Jie Gao, Yimin Yu, Jingjing Huang, Yifeng Shen, Huafang Li

**Affiliations:** ^1^Department of Psychiatry, Shanghai Mental Health Center, School of Medicine, Shanghai Jiao Tong University, Shanghai, China; ^2^Department of Psychiatry, Shanghai Pudong New Area Mental Health Center, School of Medicine, Tongji University, Shanghai, China; ^3^Yingbo Community Health Service Center, Shanghai, China; ^4^Shanghai Clinical Research Center for Mental Health, Shanghai, China; ^5^Shanghai Key Laboratory of Psychotic Disorders, Shanghai Mental Health Center, School of Medicine, Shanghai Jiaotong University, Shanghai, China

**Keywords:** miR-124, methylation, major depressive disorder, antidepressant, biomarker

## Abstract

Based on our previous studies and other evidence, miR-124 is an important biomarker and therapeutic target for major depressive disorder (MDD). The aim of this study was to clarify the role of miR-124 methylation in MDD and antidepressant effects from the perspective of epigenetics. MethylTarget™ was used to detect methylation levels of the three miR-124 precursor genes (MIR124-1, MIR124-2, and MIR124-3) in 33 pre- and post-treatment MDD patients and 33 healthy controls. A total of 11 cytosine-phosphate-guanine (CpG) islands in the three miR-124 precursor genes, including 222 CpG sites, were detected. All CpG islands were hypomethylated in MDD patients when compared to healthy controls and seven CpG regions were still identified with a statistically significant difference after Bonferroni correction. In addition, 137 of 222 CpG sites were found a statistical difference between MDD patients and controls, and 40 CpG sites were still statistically significant after Bonferroni correction. After performing the LASSO regression model, seven biomarkers with differential methylation among 40 CpG sites were identified. Mean methylation score was lower in MDD patients (*z* = −5.84, *p* = 5.16E-9). The AUC value reached 0.917 (95% CI: 0.854–0.981) to discriminate MDD and controls. No changes in methylation of the three miR-124 precursor genes were found in MDD patients following antidepressant treatment. The methylation of miR-124 could be a promising diagnostic biomarker for MDD.

## Introduction

MicroRNAs (miRNAs) are a class of small non-coding RNAs about 20–22 nucleotides that regulate gene expression at the post-transcriptional level (Flynt and Lai, 2008). miRNAs are abundant in the nervous system and can regulate two-thirds of genes in the human brain (Krol et al., 2010). Among miRNAs, miR-124 is specifically expressed in the central nervous system and is one of the most abundant miRNAs in the adult brain (Lagos-Quintana et al., 2002). Many studies have found that miR-124 regulated some important genes for major depressive disorder (MDD) such as cyclic AMP-responsive element-binding protein 1 (CREB1) (Yang et al., 2020), specificity protein 1 (SP1), DNA damage-inducible transcript 4 (DDIT4) (Wang et al., 2018), AKT1 substrate 1 (AKT1S1), nuclear receptor subfamily 3 group C member 1 (NR3C1) (Roy et al., 2017), and mitogen-activated protein kinase 14 (MAPK14) (Liu et al., 2018). It also regulated some critical pathways, such as the mammalian target of rapamycin (mTOR) (Wang et al., 2018), glucocorticoid receptor (Wang et al., 2017), and the brain-derived neurotrophic factor (BDNF)-TrkB signaling pathways (Bahi et al., 2014).

Many studies showed that miR-124 was abnormally expressed in animal models with depression (Cao et al., 2013), in brains and peripheral blood of MDD patients (Roy et al., 2017). For example, a previous study found that miR-124 expression levels were up-regulated in the hippocampus of depression model rats induced by chronic unpredictable stress (Cao et al., 2013). A similar pattern of upregulation was also noted in the prefrontal cortex (PFC) of depressed rats (Dwivedi et al., 2015; Roy et al., 2017), in the postmortem brain and serum of MDD subjects (Roy et al., 2017). In our previous study, quantitative real-time PCR was used to detect the expression levels of miR-124 in 30 controls and 32 pre- and post-treatment MDD patients. We found that the expression level of miR-124 from peripheral blood mononuclear cells (PBMCs) in MDD patients was significantly increased than those in healthy controls, and it was significantly reduced after 8 weeks of antidepressant treatment (He et al., 2016). A recent review suggested that miR-124 was probably an important biomarker and therapeutic target for MDD (Dwivedi, 2017).

Based on our previous studies and the above evidence, we wanted to further explore the regulation mechanisms which caused the abnormalities of miR-124 expression. MiR-124 is derived from the three genes in the human genome, including MIR124-1 (8p23.1), MIR124-2 (8q12.3), and MIR124-3 (20q13.33). Our previous study didn’t find the significant difference in the distribution of allelic and genotypic frequencies of rs531564 [a functional single nucleotide polymorphism (SNP) in MIR124-1 gene] between MDD patients and controls (Zeng et al., 2018). Therefore, we speculated that functional SNP in MIR124-1 gene might be unrelated to the pathophysiological mechanism of MDD. Epigenetic modifications, including cytosine-phosphate-guanine (CpG) methylation, regulation by non-coding RNAs, and histone modifications, influence gene expression without changing the DNA sequence (Goldberg et al., 2007). Increasing evidence has elucidated the methylation-dependent regulation of miRNA expression (Cai et al., 2009). The expressions of miRNA genes, especially those located near CpG islands, tend to be influenced readily by methylation (Lujambio et al., 2007; Lehmann et al., 2008; Cai et al., 2009).

To our knowledge, only one animal study has explored the association between miR-124 methylation and depression. The miR-124-3 promoter was found to be hypomethylated in PFC (∼0.7-fold less enrichment) of depressed rats (chronic exogenous corticosterone-treated, CORT-treated) compared to the control group, and the expression of Dnmt3a gene was significantly reduced in CORT-treated rats without any changes in Dnmt1 and Dnmt3b (Roy et al., 2017). However, whether the methylation of miR-124 is differentially expressed in patients with MDD and is associated with antidepressant treatment is still unclear.

Therefore, we hypothesized that the methylation of the three precursor genes of miR-124 (MIR124-1, MIR124-2, and MIR 124-3) might be related to MDD and antidepressant response. On the basis of the previous finding, the aim of the present study was to further clarify the role of miR-124 in pathophysiological mechanisms of MDD and in antidepressant effects from the perspective of epigenetics.

## Materials and Methods

### Participants

A total of 33 MDD patients were collected from the Shanghai Mental Health Center, Shanghai Jiao Tong University School of Medicine. All diagnoses were made by trained psychiatrists according to the criteria of the *Diagnostic and Statistical Manual of Mental Disorders, Fourth Edition* (*DSM-IV*) Axis-I Disorders. The inclusion criteria were as follows: (1) age 18–65 years, (2) Han ethnicity, (3) Hamilton Rating Scale for Depression (HAMD-17) > 17, and (4) either medication-free for at least 8 weeks before recruitment or medication-naïve. With the exception of MDD, patients with any other Axis-I psychiatric disorders were excluded from this study. Pregnant or lactating participants were also excluded. A total of 33 healthy volunteers of Han ethnicity matched in age and sex were recruited as controls. Subjects with severe physical illness or any history of Axis-I mental illness or whose first-degree relatives with psychiatric disorders were not included. This study was approved by the Institutional Review Board of Shanghai Mental Health Center, and all participants signed informed consent.

### Treatment and Follow-Up

Of the 33 patients initially enrolled, three MDD patients completed 1-week follow-up, three completed 2-week follow-up, four completed 4-week follow-up, and two completed 6-week follow-up, the remaining 21 MDD patients completed 8 weeks of antidepressant treatment. Patients were treated with several antidepressants, including paroxetine (*N* = 11), duloxetine (*N* = 15), and venlafaxine (*N* = 7). All drugs in this study were treated with monotherapy. Using the HAMD-17, the Montgomery–Åsberg Depression Rating Scale (MADRS), Hamilton Anxiety Scale (HAMA), and the clinical global impression (CGI) scale, the clinical symptoms of the patients were assessed regularly at baseline (week 0), week 1, week 2, week 4, week 6, and week 8. Responder was defined as a reduction in HAMD-17 score of at least 50% at week 8, while the remitter was defined as having a HAMD-17 score ≤ 7.

### DNA Extraction and CpG Sites Selection

**FIGURE 1:**
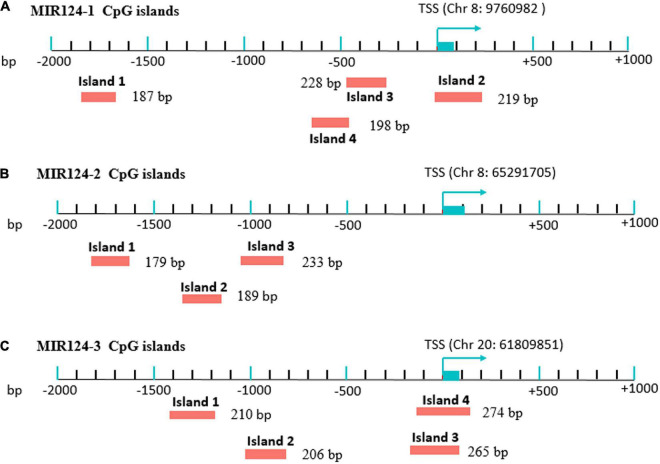
Eleven CpG islands in the promoters of the three miR-124 precursor genes. **(A)** Four islands in the MIR124-1 gene (MIR124-1_1, MIR124-1_2, MIR124-1_3 and MIR124-1_4). **(B)** Three islands in the MIR124-2 gene (MIR124-2_1, MIR124-2_2 and MIR124-2_3). **(C)** Four islands in the MIR124-3 gene (MIR124-3_1, MIR124-3_2, MIR124-3_3, and MIR124-3_4). CpG islands were selected from 2 k upstream of transcriptional start site (TSS) to 1 k downstream.

Genomic DNA was extracted from peripheral blood of MDD patients and controls using a Tiangen DNA isolation kit (Tiangen Biotech, Inc., Beijing, China) according to the manufacturer’s instructions and was stored at −80°C before the DNA methylation. Methylation analysis was performed at a concentration of 20 ng/μL. CpG islands located in the promoters of the three miR-124 precursor genes (MIR124-1, MIR124-2, and MIR124-3) were selected according to the following criteria from 2 k upstream of transcriptional start site (TSS) to 1 k downstream of the first exon: (1) Observed/Expected dinucleotides CpG ratio > 0.60; (2) Cytosine-Guanine content > 50%; and (3) Length > 200 bp. Finally, we selected 11 CpG regions of the three miR-124 precursor gene promoters including 222 CpG sites (Figure 1). The details of the CpG regions are listed in Table 1.

**TABLE 1:** Details of the CpG regions of miR-124.

Target	Chr	Gene	TSS	Start	End	Length	Target_Strand	Distance2TSS
MIR124-1_1	8	MIR124-1	9760982	9762839	9762653	187	−	−1857
MIR124-1_2	8	MIR124-1	9760982	9760765	9760983	219	+	217
MIR124-1_3	8	MIR124-1	9760982	9761472	9761245	228	−	−490
MIR124-1_4	8	MIR124-1	9760982	9761467	9761664	198	+	−485
MIR124-2_1	8	MIR124-2	65291705	65289879	65290057	179	+	−1826
MIR124-2_2	8	MIR124-2	65291705	65290522	65290334	189	−	−1183
MIR124-2_3	8	MIR124-2	65291705	65290874	65290642	233	−	−831
MIR124-3_1	20	MIR124-3	61809851	61808446	61808655	210	+	−1405
MIR124-3_2	20	MIR124-3	61809851	61809036	61808831	206	−	−815
MIR124-3_3	20	MIR124-3	61809851	61809667	61809931	265	+	−184
MIR124-3_4	20	MIR124-3	61809851	61809699	61809972	274	+	−152

*Chr, chromosome; TSS, the transcription start site; Target_Strand, the direction of product; Distance2TSS, the distance from the product to the TSS.*

### DNA Methylation Detection

The DNA methylation levels were determined by MethylTarget™ (Genesky Biotechnologies Inc.), which used the next-generation sequencing technology for multiple targeted CpG methylation analysis. Genomic DNA (400 ng) was treated with sodium bisulfite using EZ DNA Methylation™-GOLD Kit (ZYMO RESEARCH, CA, United States) according to manufacturer’s protocols. Optimized primer set combinations were used for multiplex PCR (Table 2). PCR amplicons were separated by agarose electrophoresis and purified using QIAquick Gel Extraction kit (QIAGEN, Hilden, Germany). After the library construction, samples were sequenced using the paired-end sequencing protocol on the Illumina Hiseq/Miseq platform according to the manufacturer’s guidelines. The methylation level of each CpG site was obtained as the percentage of methylated cytosine in total cytosine. The methylation status of the promoter region and the first exon was defined as the average methylation level of all CpG sites in the region.

**TABLE 2:** Primers used for the MethylTarget assays.

Target	PrimerF	PrimerR
MIR124-1_1	TGGGGATTGYGGT TAGTTTTT	AAAACAATCCRAATA CCCTCAAC
MIR124-1_2	GGATAGGTAGTYGGAG GGAGTTTTAG	AAAACCTCTCTCTCC RTATTCACAA
MIR124-1_3	TTGGGYGAAGTAGAG GGYGATA	TTCRCATCCAAACTTT TTCCTACA
MIR124-1_4	GTTTAGGAAATTGA AGGGGATTAGG	TAAATCCCCAAAT CCCAACC
MIR124-2_1	GGATTAGTAAGYGA GGGGGAAAGA	CTACTAACCCTATTCAAAC CTACAAAA
MIR124-2_2	GGATATAAGGTGATAAGAG GATAGGATTTT	CAACRCATACAC CCTCCTCTAAAC
MIR124-2_3	AGTGGTTATAGTTTG GGGTTGGA	AACCAACTCTCAACTCTC TCTCCT
MIR124-3_1	GTGYGTTTTGTAGGYGT TAGATAGGAG	CACACTCCAAC CCCTCACA
MIR124-3_2	YGTTGTAGGYGTTTAG TTTTTGTTAGTT	CCCCRCAATTCTCA AAAACA
MIR124-3_3	GGAGAAGTGTGGGT TTTTTYGAGT	CCTCTCTTAACATTCA CCRCRTACC
MIR124-3_4	GATTGGGATAGTATAGT YGGTTGAGYG	AACTCRCRAACCAT TTCCATAAAA

### Statistical Analyses

Chi-square tests were used to assess the categorical variables such as gender. The Shapiro-Wilk test was used to assess the normal distribution of the data. Independent *T*-test or Mann–Whitney *U*-test was used to analyze the methylation levels of CpG islands or CpG sites between MDD and controls. The paired-sample *T*-test or Wilcoxon signed-rank test was used to analyze the methylation levels of CpG islands or CpG sites before and after antidepressant treatment. Bonferroni correction was performed by multiplying the unadjusted *P*-values by the number of the CpG islands or CpG sites. Last Observation Carried Forward (LOCF) imputation was used on the intention-to-treat population, where the last unmissed observation was carried forward from the time of withdrawal to the end of the study. These statistical analyses were performed by SPSS 20.0 software (SPSS Inc., Chicago, IL, United States).

The LASSO logistic regression analysis was used to identify the candidate CpG sites that were most informative and to omit the CpG sites which contributed little to the model. LASSO is a commonly used regression method of high-dimensional variables reduction and can shrink the estimated coefficients by tuning parameter lambda (λ) (Tibshirani, 1996). In this study, LASSO was performed for CpG sites that remained significantly different after Bonferroni correction between MDD and controls. Bootstrap resampling, which has been demonstrated to produce stable, almost unbiased estimates of predictive accuracy and is more efficient than other methods (Steyerberg et al., 2001), was conducted for the internal validation to validate the predictive performance of the current diagnostic model. Therefore, bootstrap resampling method with 1,000-iteration resampling was used to draw the 33 MDD patients and 33 controls for the validation dataset. Receiver operating characteristic (ROC) curve and area under the curve (AUC) were used to evaluate the discriminating ability and the diagnostic performance of the model. Calibration curve was plotted for visualizing the model prediction accuracy. The R packages, such as “glmnet,” “pROC,” “ggplot2,” and “rms,” were performed in this study. A two-tailed value of *P* < 0.05 was defined significant after Bonferroni correction.

## Results

### Demographic and Clinical Characteristics

The demographic and clinical characteristics of the participants are listed in Table 3. There were no significant differences in gender, age, and body mass index (BMI) between MDD patients and healthy controls.

**TABLE 3:** Demographic and clinical characteristics of the subjects.

	MDD (*n* = 33)	Controls (*n* = 33)	χ^2^ or *t* or *z*	*p*-value
Sex (M/F)	18/15	14/19	0.971	0.325^a^
^c^Age (years)	33.76 ± 12.48	34.91 ± 11.85	−0.019	0.985^b^
^c^BMI	23.22 ± 3.34	22.50 ± 2.92	−0.679	0.497^b^
^c^Duration of current episode (months)	11.28 ± 13.98			
^c^Duration of illness (months)	29.33 ± 4.48			
^c^Baseline HAMD-17 scores	22.64 ± 2.66			
^c^Baseline HAMA scores	16.33 ± 4.81			
^c^Baseline CGI scores	4.55 ± 0.75			

*MDD, major depressive disorder; BMI, body mass index; HAMD-17, 17-Item Hamilton Rating Scale for Depression; HAMA, Hamilton Anxiety Scale; CGI, clinical global impression.*

*^a^Chi-square test.*

*^b^Mann–Whitney test.*

*^c^Mean ± standard deviation (SD).*

### Lower Methylation Levels of the Three miR-124 Precursor Genes in Major Depressive Disorder Patients

**FIGURE 2:**
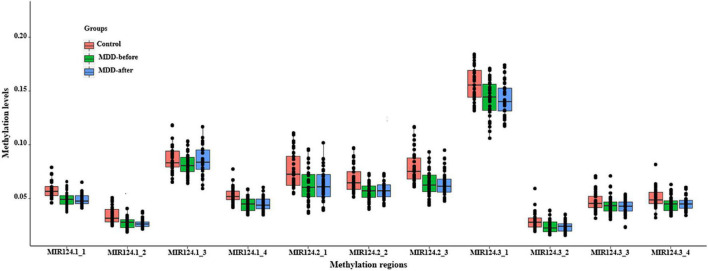
The methylation levels of the 11 CpG regions in three miR-124 precursor genes. All CpG islands were hypomethylated in MDD patients when compared to healthy controls.

Based on the CpG islands adjacent to the promoter regions of the three miR-124 precursor genes, 11 CpG regions (MIR124-1_1, MIR124-1_2, MIR124-1_3, MIR124-1_4, MIR124-2_1, MIR124-2_2, MIR124-2_3, MIR124-2_3, MIR124-3_1, MIR124-3_2, MIR124-3_3, and MIR124-3_4, the details of which can be found in Table 1) were amplified and sequenced. The result demonstrated that there was a lower methylation pattern in MDD patients when compared to healthy controls, nine CpG regions were identified with a statistically significant difference, and seven CpG regions remained significant after Bonferroni correction (Table 4 and Figure 2).

**TABLE 4:** Methylation levels of the 11 CpG regions in three miR-124 precursor genes between MDD patients and controls.

Target	Normal (Mean ± SD)	MDD patients (mean ± SD)	*t* or *z*	*p*-value	Corrected *p*-value^b^
MIR124-1_1	0.058 ± 0.008	0.049 ± 0.007	−4.28	9.25E-06^a^	**1.02E-04**
MIR124-1_2	0.034 ± 0.007	0.027 ± 0.005	−4.01	3.05E-05^a^	**3.36E-04**
MIR124-1_3	0.087 ± 0.012	0.084 ± 0.014	−0.92	0.360	N.S.
MIR124-1_4	0.053 ± 0.008	0.045 ± 0.007	−3.95	3.93E-05^a^	**4.32E-04**
MIR124-2_1	0.076 ± 0.017	0.061 ± 0.016	−3.37	3.76E-04^a^	**4.14E-03**
MIR124-2_2	0.068 ± 0.012	0.056 ± 0.008	−4.11	1.94E-05^a^	**2.13E-04**
MIR124-2_3	0.079 ± 0.015	0.064 ± 0.013	−3.87	5.37E-05^a^	**5.91E-04**
MIR124-3_1	0.158 ± 0.02	0.144 ± 0.021	−2.51	0.006^a^	0.066
MIR124-3_2	0.029 ± 0.008	0.024 ± 0.006	−3.00	0.001^a^	**0.011**
MIR124-3_3	0.047 ± 0.009	0.044 ± 0.008	−1.40	0.081^a^	0.891
MIR124-3_4	0.051 ± 0.009	0.045 ± 0.007	−2.57	0.005^a^	0.055

*N.S., non-significant.*

*^a^Mann–Whitney test.*

*^b^Calculated by Bonferroni correction.*

*Bold numbers represent significant p-value.*

In addition, the methylation level of each CG site was assessed and 137 of 222 CpG sites in the three miR-124 precursor genes were found a statistical difference between MDD patients and controls (Supplementary Table 1). Among them, 40 CpG sites were still statistically significant after Bonferroni correction (Supplementary Table 1).

### Dimensionality Reduction and Model Construction

**FIGURE 3:**
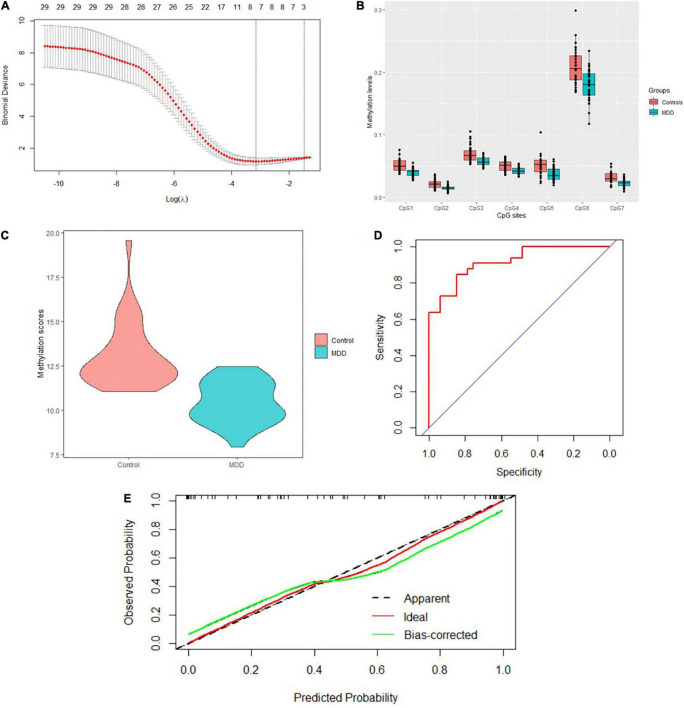
Model construction and the internal validation. **(A)** The left dashed line indicated the optimal lambda [log (λ) = –3.15]. **(B)** Methylation levels of the seven CpG sites were significantly lower in MDD patients compared to healthy controls. **(C)** Mean methylation score was also lower in MDD patients compared to controls. **(D)** The AUC value reached 0.917 (95% CI: 0.854–0.981) using the methylation score to discriminate MDD and controls. **(E)** Calibration curve showed the predictive ability of the model for depression and controls. The closer the distance between the solid and dashed lines, the better the predictive power of the selected model (*B* = 1,000).

A total of 40 differentially expressed CpG sites between MDD and controls were performed for the LASSO regression analysis. The optimal λ was selected for the cross-validation error at the minimum value, which is 0.04 [log (λ) = −3.15] (Figure 3A). At this value of lambda, seven biomarkers with differential methylation (CpG 1–7) were identified (Table 5). The methylation levels of all these seven CpG biomarkers were significantly lower in MDD patients compared to controls (Table 5 and Figure 3B), and the significance remained after Bonferroni correction. Then, these seven CpG sites were used to construct the model based on coefficients. The methylation score of each subject was calculated with the absolute value of non-zero coefficients (all non-zero coefficients were negative) by the following equation, taking into consideration of individual methylation level for each of these seven CpG sites:


$$\begin{array}{l} \text{Methylation score}=(\text{52}.\text{14}\times \text{CpG1})+(\text{53}.\text{18}\\ \;\;\;\;\;\;\;\;\;\;\;\;\times \text{CpG2})+(\text{45}.\text{28}\times \text{CpG3})+\\ \;\;\;\;\;\;\;\;\;\;\;\;(\text{33}.\text{85}\times \text{CpG4})+(\text{12}.\text{42}\times \\ \;\;\;\;\;\;\;\;\;\;\;\;\text{CpG5})+(\text{13}.\text{1}0\times \text{CpG6})\\ \;\;\;\;\;\;\;\;\;\;\;\;+(\text{34}.\text{95}\times \text{CpG 7}). \end{array}$$

**TABLE 5:** Methylation levels of seven screened CpG sites between MDD patients and controls.

ID	Target	Position	Non-zero coefficient	Normal	MDD patients	*t* or *z*	*p*-value	Corrected *p*-value^b^
CpG 1	MIR124-1_4	94	−52.14	0.050 ± 0.009	0.040 ± 0.006	−4.48	3.68E-06^a^	**8.17E-04**
CpG 2	MIR124-1_2	46	−53.18	0.021 ± 0.006	0.015 ± 0.004	−4.22	1.22E-05^a^	**2.70E-03**
CpG 3	MIR124-2_2	42	−45.28	0.069 ± 0.013	0.057 ± 0.007	−4.75	1.95E-05^a^	**4.33E-03**
CpG 4	MIR124-1_1	123	−33.85	0.050 ± 0.009	0.042 ± 0.005	−3.89	5.05E-05^a^	**0.011**
CpG 5	MIR124-3_2	108	−12.42	0.069 ± 0.013	0.057 ± 0.007	−4.11	1.94E-05	**4.31E-03**
CpG 6	MIR124-3_1	92	−13.10	0.210 ± 0.029	0.179 ± 0.025	−3.93	4.19E-05^a^	**9.30E-03**
CpG 7	MIR124-3_2	34	−34.95	0.031 ± 0.029	0.023 ± 0.023	−4.06	1.47E-04	**0.033**

*^a^Mann–Whitney test.*

*^b^Corrected p-value was the p-value multiplied by 222.*

*Bold numbers represent significant p-value.*

The mean methylation score was also lower in MDD patients compared to controls (10.46 ± 1.18 vs. 13.05 ± 1.82, *z* = −5.84, *p* = 5.16E-9) (Figure 3C). The AUC value reached 0.917 (95% CI: 0.854–0.981) using the methylation score to discriminate MDD and controls (Figure 3D). Bootstrap resampling (*n* = 1000) was used for the internal validation of the diagnostic model. The model showed a good discrimination (bias-corrected AUC = 0.872, 95%CI = 0.871–0.874). The calibration curve showed good agreement between the constructed model’s prediction of MDD patients and the true observation (Figure 3E).

### No Changes in Methylation of the Three miR-124 Precursor Genes in Major Depressive Disorder Patients Following Treatment

Hamilton Rating Scale for Depression-17 scores and the methylation levels of miR-124 gene before and after treatment were analyzed in 33 MDD patients. The HAMD-17 scores in the 33 patients were significantly decreased after antidepressant treatment compared to those at baseline (22.64 ± 2.66 vs. 12.45 ± 7.95, *z* = −4.55, *p* < 0.001). There were no significant differences in methylation levels of the 11 CpG regions after treatment (Supplementary Table 2). In addition, the methylation levels of two CpG sites (position 63: 0.032 ± 0.017 vs. 0.024 ± 0.015, *z* = −1.94, *p* = 0.026; position 94: 0.027 ± 0.013 vs. 0.017 ± 0.012, *t* = 3.19, *p* = 0.002) located in the MIR124-3_3 island were significantly lower after treatment while the other 220 CpG sites showed no changes (Supplementary Table 3). However, the significance disappeared after Bonferroni correction.

Likewise, 21 MDD patients who completed 8 weeks of antidepressant treatment were retained for sensitivity analysis. We found that the HAMD-17 scores in these 21 MDD patients were still significantly decreased at the end of the 8 weeks of treatment compared to baseline levels (23.1 ± 2.74 vs. 9.62 ± 6.58, *z* = −3.98, *p* < 0.001). The methylation level of MIR124-1_3 CpG region was higher after treatment (0.081 ± 0.01 vs. 0.091 ± 0.018, *t* = −2.27, *p* = 0.034, correction *p* = 0.374), and the other 10 CpG islands showed no significant changes. Among the 222 CpG sites, the methylation level of position 94 CpG site (0.026 ± 0.014 vs. 0.017 ± 0.010, *t* = 2.49, *p* = 0.022) was still significantly lower after antidepressant treatment. However, the significance disappeared after Bonferroni correction.

## Discussion

To our best knowledge, this is the first study to explore the methylation levels of the three miR-124 precursor genes between MDD patients and healthy controls. In our study, seven CpG islands and 40 CpG sites in the promoter regions of the miR-124 were identified with a statistically significant difference after Bonferroni correction between MDD and controls. A diagnostic model was also constructed using Lasso regression among 40 CpG sites and the internal validation analysis showed that the model has a good ability to distinguish MDD from healthy controls. In addition, a large number of studies have found that methylation of miRNA could be a useful biomarker for the diagnosis of disease (Shimizu et al., 2013; Torres-Ferreira et al., 2017; Loginov et al., 2018; Rogeri et al., 2018). Therefore, we speculated that miR-124 methylation might be a diagnostic biomarker for MDD.

To date, only one animal study has explored the relationship between miR-124 methylation and depression. Roy et al. (2017) used methylated DNA immunoprecipitation (MeDIP) assay to detect the CpG islands within 1 KB upstream of the transcription start site in the promoter region of pre-miR-124-3 (chromosome 3) in rats and found that the miR-124-3 promoter in depressed rats (CORT-treated) was hypomethylated in the PFC compared to the control group (Roy et al., 2017). Although species (human vs. rat) are different, our study is consistent with the findings of Roy et al. (2017) that miR-124 methylation is lower in the state of depression. Tseng et al. (2014) also found a decrease in global DNA methylation in patients with severe depression than in healthy controls. In addition, as mentioned above, miR-124 methylation could also be used as a diagnostic biomarker in tumor tissues. However, hypermethylation was predominant in tumors, while hypomethylation was predominant in MDD, which might be related to the disease specificity as well as the temporal and spatial specificity of miRNA expression (Plasterk, 2006).

CpG methylation is an important regulatory mechanism of gene expression, abnormal promoter methylation can lead to abnormal activation or silencing of genes (Manton et al., 2005; Wu et al., 2005). Hypermethylation and hypomethylation of neighboring CpG islands can repress and activate miRNA genes (Cai et al., 2009). Our previous study found that the expression level of miR-124 from PBMCs in MDD patients was significantly increased than those in healthy controls (He et al., 2016). Similar upregulation patterns have been found not only in the postmortem brain and serum of MDD subjects (Dwivedi et al., 2015; Roy et al., 2017) but also in the PFC of depressed rats (Roy et al., 2017). In this study, we found that all CpG islands were hypomethylated in MDD patients and seven CpG regions were identified with a statistically significant difference after Bonferroni correction. Therefore, we speculated that the hypomethylation of the three miR-124 precursor genes might be one of the reasons for the high gene expression of miR-124.

In this study, no changes in methylation of the three miR-124 precursor genes were found in MDD patients following antidepressant treatment. There may be several reasons as follows. First, based on our total sample of 33 individuals, a power analysis for samples pre- and post-treatment was carried out by the G* Power program (Erdfelder et al., 1996). The sample size had a *post hoc* power of 0.050–0.315 for the 11 CpG islands of the three miR-124 precursor gene at the 0.05 significance level. Therefore, small sample size and small power value were insufficient for longitudinal cohort study. A larger sample size may be required to illustrate more significant *p*-values. Second, the duration of treatment at 8 weeks was relatively short, and it may be difficult to cause changes for miR-124 methylation levels. Many studies also showed no significant difference in methylation levels at 8 weeks or longer time. For example, SLC6A4 promoter methylation status was not associated with treatment outcomes after a 12-week treatment with antidepressants in 108 MDD patients (Kang et al., 2013). Finally, it was also possible that the methylation levels of the three miR-124 precursor genes may just make only a very small or no contribution to antidepressant response. Therefore, the research findings should be verified in another study.

There were several limitations in our study. First, the sample size was relatively small. Therefore, the research findings should be verified in another study with a larger sample size. Second, the methylation and mRNA expression levels of miR-124 were not detected simultaneously in the same subjects in this study. Therefore, we could not directly find whether the upregulation of miR-124 was related to the hypomethylation of the three precursor genes of miR-124 (MIR124-1, MIR124-2, and MIR124-3). Third, considering the tissue or disease specificity of miR-124, it would be better for further study to explore the expression levels (mRNA and methylation levels) of miR-124 in other tissues, such as serum and plasmas, or other mental diseases, such as schizophrenia and bipolar disorder. Finally, some confounders were not considered in this study including economic conditions, dietary habits, social support, and lifestyle that may have impacted the methylation levels of miR-124 and antidepressant response.

## Conclusion

Our study found that the methylation levels of the three miR-124 precursor genes were significantly reduced in MDD patients. The methylation of miR-124 could be a promising diagnostic biomarker for MDD.

## Data Availability Statement

The original contributions presented in the study are included in the article/Supplementary Material; further inquiries can be directed to the corresponding authors.

## Ethics Statement

This study was approved by the Institutional Review Board of Shanghai Mental Health Center. The patients/participants provided their written informed consent to participate in this study.

## Author Contributions

DZ designed the study, ran data analysis, and wrote the manuscript. SH and NZ contributed to the experimental work and writing of the manuscript. MH, JG, and YY recruited patients and obtained their clinical information. HL, JH, and YS led data analyses and offered much constructive advice to the study. All authors participated in the design of the experiments, critical discussion of the findings, and read and approved the final manuscript.

## Conflict of Interest

The authors declare that the research was conducted in the absence of any commercial or financial relationships that could be construed as a potential conflict of interest.

## Publisher’s Note

All claims expressed in this article are solely those of the authors and do not necessarily represent those of their affiliated organizations, or those of the publisher, the editors and the reviewers. Any product that may be evaluated in this article, or claim that may be made by its manufacturer, is not guaranteed or endorsed by the publisher.
