# Supplementary material for: Promoter Hypomethylation of miR-124 Gene Is Associated With Major Depressive Disorder
Source: Front Mol Neurosci. 2021 Dec 21;14:771103. doi: 10.3389/fnmol.2021.771103 (PMC8724533; doi:10.3389/fnmol.2021.771103)
Supplement: Supplementary file 1 [file Data_Sheet_1.docx]

Table S1. The statistical results of 222 CpG sites between MDD patients and controls.

|  | **Target** | **Position** | **t or z** | **p-value** | **corrected p-value** |
| --- | --- | --- | --- | --- | --- |
| 1 | MIR124-2_3 | 158 | -4.70 | 1.29E-06 | **2.85E-04** |
| 2 | MIR124-1_1 | 136 | -4.64 | 1.75E-06 | **3.88E-04** |
| 3 | MIR124-1_2 | 67 | -5.27 | 2.07E-06 | **4.60E-04** |
| 4 | MIR124-1_4 | 82 | -4.51 | 3.18E-06 | **7.06E-04** |
| 5 | MIR124-1_4 | 94 | -4.48 | 3.68E-06 | **8.17E-04** |
| 6 | MIR124-1_2 | 40 | -4.44 | 4.57E-06 | **1.02E-03** |
| 7 | MIR124-2_2 | 146 | -4.92 | 6.71E-06 | **1.49E-03** |
| 8 | MIR124-2_2 | 133 | -4.94 | 6.86E-06 | **1.52E-03** |
| 9 | MIR124-1_1 | 159 | -4.79 | 1.04E-05 | **2.31E-03** |
| 10 | MIR124-1_2 | 46 | -4.22 | 1.22E-05 | **2.70E-03** |
| 11 | MIR124-1_4 | 148 | -4.21 | 1.30E-05 | **2.89E-03** |
| 12 | MIR124-2_2 | 34 | -4.16 | 1.59E-05 | **3.53E-03** |
| 13 | MIR124-2_1 | 36 | -4.15 | 1.69E-05 | **3.75E-03** |
| 14 | MIR124-1_1 | 148 | -4.69 | 1.70E-05 | **3.77E-03** |
| 15 | MIR124-3_2 | 108 | -4.11 | 1.94E-05 | **4.31E-03** |
| 16 | MIR124-2_2 | 42 | -4.75 | 1.95E-05 | **4.33E-03** |
| 17 | MIR124-2_3 | 146 | -4.64 | 1.95E-05 | **4.33E-03** |
| 18 | MIR124-2_1 | 31 | -4.05 | 2.52E-05 | **5.59E-03** |
| 19 | MIR124-1_1 | 84 | -4.02 | 2.86E-05 | **6.36E-03** |
| 20 | MIR124-2_2 | 94 | -3.96 | 3.69E-05 | **8.20E-03** |
| 21 | MIR124-1_4 | 100 | -4.42 | 3.94E-05 | **8.76E-03** |
| 22 | MIR124-1_4 | 92 | -4.43 | 4.13E-05 | **9.18E-03** |
| 23 | MIR124-3_1 | 92 | -3.93 | 4.19E-05 | **9.30E-03** |
| 24 | MIR124-2_2 | 108 | -4.38 | 4.44E-05 | **9.85E-03** |
| 25 | MIR124-1_1 | 123 | -3.89 | 5.05E-05 | **0.011** |
| 26 | MIR124-1_4 | 96 | -4.32 | 5.71E-05 | **0.013** |
| 27 | MIR124-1_1 | 164 | -3.84 | 6.07E-05 | **0.013** |
| 28 | MIR124-1_2 | 50 | -3.84 | 6.07E-05 | **0.013** |
| 29 | MIR124-2_3 | 161 | -3.83 | 6.45E-05 | **0.014** |
| 30 | MIR124-1_1 | 152 | -4.30 | 6.90E-05 | **0.015** |
| 31 | MIR124-2_3 | 134 | -4.25 | 7.54E-05 | **0.017** |
| 32 | MIR124-2_3 | 106 | -4.20 | 8.56E-05 | **0.019** |
| 33 | MIR124-1_2 | 44 | -3.74 | 9.24E-05 | **0.021** |
| 34 | MIR124-1_2 | 57 | -4.12 | 1.13E-04 | **0.025** |
| 35 | MIR124-2_3 | 149 | -3.66 | 1.24E-04 | **0.027** |
| 36 | MIR124-1_1 | 125 | -3.64 | 1.39E-04 | **0.031** |
| 37 | MIR124-3_2 | 34 | -4.06 | 1.47E-04 | **0.033** |
| 38 | MIR124-1_4 | 120 | -3.61 | 1.56E-04 | **0.035** |
| 39 | MIR124-2_1 | 26 | -3.55 | 1.95E-04 | **0.043** |
| 40 | MIR124-1_1 | 155 | -3.98 | 1.96E-04 | **0.044** |
| 41 | MIR124-1_4 | 144 | -3.86 | 2.76E-04 | 0.061 |
| 42 | MIR124-1_1 | 131 | -3.84 | 2.85E-04 | 0.063 |
| 43 | MIR124-2_1 | 87 | -3.84 | 2.92E-04 | 0.065 |
| 44 | MIR124-2_2 | 38 | -3.41 | 3.20E-04 | 0.071 |
| 45 | MIR124-2_2 | 74 | -3.40 | 3.38E-04 | 0.075 |
| 46 | MIR124-1_1 | 97 | -3.79 | 3.44E-04 | 0.076 |
| 47 | MIR124-2_2 | 161 | -3.78 | 3.50E-04 | 0.078 |
| 48 | MIR124-1_4 | 103 | -3.38 | 3.57E-04 | 0.079 |
| 49 | MIR124-2_1 | 117 | -3.38 | 3.57E-04 | 0.079 |
| 50 | MIR124-2_3 | 191 | -3.75 | 3.94E-04 | 0.088 |
| 51 | MIR124-2_3 | 125 | -3.75 | 4.00E-04 | 0.089 |
| 52 | MIR124-1_2 | 55 | -3.75 | 4.17E-04 | 0.093 |
| 53 | MIR124-2_3 | 172 | -3.68 | 4.82E-04 | 0.107 |
| 54 | MIR124-2_3 | 131 | -3.67 | 5.37E-04 | 0.119 |
| 55 | MIR124-1_2 | 83 | -3.27 | 5.43E-04 | 0.121 |
| 56 | MIR124-2_3 | 203 | -3.27 | 5.43E-04 | 0.121 |
| 57 | MIR124-3_2 | 138 | -3.27 | 5.43E-04 | 0.121 |
| 58 | MIR124-1_4 | 69 | -3.25 | 5.72E-04 | 0.127 |
| 59 | MIR124-1_2 | 87 | -3.24 | 6.02E-04 | 0.134 |
| 60 | MIR124-2_2 | 51 | -3.57 | 6.93E-04 | 0.154 |
| 61 | MIR124-3_4 | 168 | -3.18 | 7.37E-04 | 0.164 |
| 62 | MIR124-3_1 | 111 | -3.15 | 8.15E-04 | 0.181 |
| 63 | MIR124-3_2 | 101 | -3.12 | 9.00E-04 | 0.200 |
| 64 | MIR124-1_4 | 176 | -3.47 | 9.39E-04 | 0.209 |
| 65 | MIR124-2_1 | 123 | -3.11 | 9.46E-04 | 0.210 |
| 66 | MIR124-1_2 | 38 | -2.98 | 0.001 | 0.222 |
| 67 | MIR124-1_2 | 158 | -3.05 | 0.001 | 0.222 |
| 68 | MIR124-1_4 | 75 | -3.03 | 0.001 | 0.222 |
| 69 | MIR124-1_1 | 107 | -3.37 | 0.001 | 0.222 |
| 70 | MIR124-1_4 | 42 | -3.32 | 0.001 | 0.222 |
| 71 | MIR124-2_2 | 99 | -3.32 | 0.001 | 0.222 |
| 72 | MIR124-3_2 | 113 | -3.00 | 0.001 | 0.222 |
| 73 | MIR124-1_4 | 61 | -2.96 | 0.002 | 0.444 |
| 74 | MIR124-1_1 | 99 | -3.22 | 0.002 | 0.444 |
| 75 | MIR124-3_1 | 95 | -3.26 | 0.002 | 0.444 |
| 76 | MIR124-3_1 | 100 | -3.28 | 0.002 | 0.444 |
| 77 | MIR124-3_1 | 106 | -3.15 | 0.002 | 0.444 |
| 78 | MIR124-3_4 | 179 | -2.93 | 0.002 | 0.444 |
| 79 | MIR124-1_4 | 133 | -2.77 | 0.003 | 0.666 |
| 80 | MIR124-2_3 | 39 | -3.10 | 0.003 | 0.666 |
| 81 | MIR124-2_3 | 167 | -2.74 | 0.003 | 0.666 |
| 82 | MIR124-3_2 | 40 | -2.71 | 0.003 | 0.666 |
| 83 | MIR124-3_2 | 70 | -2.71 | 0.003 | 0.666 |
| 84 | MIR124-3_2 | 79 | -3.10 | 0.003 | 0.666 |
| 85 | MIR124-3_2 | 91 | -3.09 | 0.003 | 0.666 |
| 86 | MIR124-3_4 | 215 | -3.14 | 0.003 | 0.666 |
| 87 | MIR124-2_3 | 129 | -2.99 | 0.004 | 0.888 |
| 88 | MIR124-3_4 | 243 | -2.67 | 0.004 | 0.888 |
| 89 | MIR124-1_2 | 74 | -2.89 | 0.005 | N.S |
| 90 | MIR124-3_1 | 56 | -2.92 | 0.005 | N.S |
| 91 | MIR124-3_2 | 142 | -2.60 | 0.005 | N.S |
| 92 | MIR124-3_4 | 143 | -2.58 | 0.005 | N.S |
| 93 | MIR124-2_2 | 44 | -2.85 | 0.006 | N.S |
| 94 | MIR124-3_4 | 37 | -2.86 | 0.006 | N.S |
| 95 | MIR124-1_2 | 156 | -2.44 | 0.007 | N.S |
| 96 | MIR124-1_1 | 87 | -2.80 | 0.007 | N.S |
| 97 | MIR124-1_4 | 171 | -2.81 | 0.007 | N.S |
| 98 | MIR124-3_2 | 76 | -2.45 | 0.007 | N.S |
| 99 | MIR124-1_1 | 43 | -2.40 | 0.008 | N.S |
| 100 | MIR124-1_4 | 169 | -2.76 | 0.008 | N.S |
| 101 | MIR124-2_3 | 111 | -2.72 | 0.008 | N.S |
| 102 | MIR124-2_2 | 72 | -2.70 | 0.009 | N.S |
| 103 | MIR124-3_1 | 116 | -2.35 | 0.009 | N.S |
| 104 | MIR124-3_4 | 139 | -2.37 | 0.009 | N.S |
| 105 | MIR124-2_3 | 94 | -2.67 | 0.01 | N.S |
| 106 | MIR124-3_1 | 173 | -2.22 | 0.013 | N.S |
| 107 | MIR124-3_2 | 155 | -2.57 | 0.013 | N.S |
| 108 | MIR124-2_3 | 35 | -2.19 | 0.014 | N.S |
| 109 | MIR124-2_3 | 90 | -2.51 | 0.014 | N.S |
| 110 | MIR124-3_2 | 73 | -2.52 | 0.014 | N.S |
| 111 | MIR124-3_2 | 140 | -2.19 | 0.014 | N.S |
| 112 | MIR124-3_4 | 74 | -2.21 | 0.014 | N.S |
| 113 | MIR124-3_1 | 122 | -2.14 | 0.016 | N.S |
| 114 | MIR124-3_1 | 171 | -2.14 | 0.016 | N.S |
| 115 | MIR124-3_2 | 29 | -2.48 | 0.016 | N.S |
| 116 | MIR124-3_4 | 51 | -2.46 | 0.017 | N.S |
| 117 | MIR124-3_3 | 85 | -2.41 | 0.019 | N.S |
| 118 | MIR124-1_4 | 153 | -2.40 | 0.02 | N.S |
| 119 | MIR124-2_2 | 101 | -2.40 | 0.02 | N.S |
| 120 | MIR124-1_2 | 91 | -2.35 | 0.022 | N.S |
| 121 | MIR124-3_1 | 62 | -2.34 | 0.022 | N.S |
| 122 | MIR124-2_2 | 131 | -2.34 | 0.023 | N.S |
| 123 | MIR124-3_2 | 61 | -2.33 | 0.023 | N.S |
| 124 | MIR124-3_4 | 56 | -2.33 | 0.023 | N.S |
| 125 | MIR124-3_2 | 119 | -1.98 | 0.024 | N.S |
| 126 | MIR124-1_4 | 139 | -2.29 | 0.026 | N.S |
| 127 | MIR124-3_1 | 104 | -1.89 | 0.03 | N.S |
| 128 | MIR124-3_4 | 111 | -1.84 | 0.033 | N.S |
| 129 | MIR124-3_2 | 182 | -1.83 | 0.034 | N.S |
| 130 | MIR124-3_1 | 120 | -1.81 | 0.035 | N.S |
| 131 | MIR124-3_4 | 46 | -2.14 | 0.036 | N.S |
| 132 | MIR124-3_2 | 123 | -1.76 | 0.04 | N.S |
| 133 | MIR124-3_3 | 168 | -2.09 | 0.04 | N.S |
| 134 | MIR124-3_4 | 62 | -1.76 | 0.04 | N.S |
| 135 | MIR124-1_1 | 144 | -1.73 | 0.042 | N.S |
| 136 | MIR124-2_1 | 133 | -1.73 | 0.042 | N.S |
| 137 | MIR124-1_3 | 105 | -2.06 | 0.044 | N.S |
| 138 | MIR124-3_2 | 168 | -1.62 | 0.052 | N.S |
| 139 | MIR124-3_1 | 87 | -1.89 | 0.064 | N.S |
| 140 | MIR124-3_4 | 136 | -1.50 | 0.066 | N.S |
| 141 | MIR124-3_4 | 213 | -1.50 | 0.066 | N.S |
| 142 | MIR124-3_2 | 55 | -1.86 | 0.068 | N.S |
| 143 | MIR124-3_2 | 57 | -1.49 | 0.068 | N.S |
| 144 | MIR124-3_3 | 130 | -1.49 | 0.068 | N.S |
| 145 | MIR124-2_1 | 143 | -1.83 | 0.072 | N.S |
| 146 | MIR124-1_2 | 105 | -1.80 | 0.077 | N.S |
| 147 | MIR124-1_4 | 161 | -1.41 | 0.079 | N.S |
| 148 | MIR124-3_4 | 234 | -1.77 | 0.081 | N.S |
| 149 | MIR124-3_4 | 53 | -1.77 | 0.083 | N.S |
| 150 | MIR124-1_2 | 123 | -1.35 | 0.088 | N.S |
| 151 | MIR124-3_3 | 164 | -1.73 | 0.089 | N.S |
| 152 | MIR124-2_3 | 185 | -1.71 | 0.092 | N.S |
| 153 | MIR124-1_1 | 63 | -1.31 | 0.096 | N.S |
| 154 | MIR124-3_1 | 39 | -1.69 | 0.096 | N.S |
| 155 | MIR124-3_1 | 127 | -1.67 | 0.1 | N.S |
| 156 | MIR124-3_2 | 67 | -1.28 | 0.101 | N.S |
| 157 | MIR124-3_2 | 82 | -1.28 | 0.101 | N.S |
| 158 | MIR124-3_3 | 211 | -1.66 | 0.101 | N.S |
| 159 | MIR124-3_2 | 64 | -1.65 | 0.103 | N.S |
| 160 | MIR124-3_3 | 175 | -1.26 | 0.105 | N.S |
| 161 | MIR124-3_1 | 50 | -1.64 | 0.107 | N.S |
| 162 | MIR124-3_4 | 83 | -1.59 | 0.117 | N.S |
| 163 | MIR124-1_2 | 116 | -1.18 | 0.118 | N.S |
| 164 | MIR124-1_3 | 158 | -1.19 | 0.118 | N.S |
| 165 | MIR124-3_4 | 240 | -1.57 | 0.12 | N.S |
| 166 | MIR124-3_3 | 88 | -1.57 | 0.122 | N.S |
| 167 | MIR124-3_1 | 29 | -1.56 | 0.123 | N.S |
| 168 | MIR124-3_4 | 31 | -1.54 | 0.129 | N.S |
| 169 | MIR124-3_3 | 102 | -1.53 | 0.13 | N.S |
| 170 | MIR124-2_2 | 115 | -1.47 | 0.147 | N.S |
| 171 | MIR124-3_2 | 171 | -1.04 | 0.148 | N.S |
| 172 | MIR124-1_2 | 113 | -1.33 | 0.189 | N.S |
| 173 | MIR124-3_1 | 77 | -0.84 | 0.201 | N.S |
| 174 | MIR124-3_1 | 169 | -0.84 | 0.201 | N.S |
| 175 | MIR124-3_4 | 76 | -0.84 | 0.201 | N.S |
| 176 | MIR124-3_4 | 106 | -1.24 | 0.219 | N.S |
| 177 | MIR124-1_3 | 141 | -0.77 | 0.22 | N.S |
| 178 | MIR124-3_4 | 117 | -0.77 | 0.22 | N.S |
| 179 | MIR124-2_3 | 26 | -1.21 | 0.231 | N.S |
| 180 | MIR124-1_3 | 41 | -0.70 | 0.241 | N.S |
| 181 | MIR124-3_3 | 171 | -1.18 | 0.242 | N.S |
| 182 | MIR124-3_1 | 70 | -0.66 | 0.256 | N.S |
| 183 | MIR124-1_2 | 193 | -0.62 | 0.266 | N.S |
| 184 | MIR124-3_3 | 63 | -0.62 | 0.266 | N.S |
| 185 | MIR124-3_3 | 106 | -0.53 | 0.299 | N.S |
| 186 | MIR124-2_1 | 121 | -0.49 | 0.313 | N.S |
| 187 | MIR124-1_1 | 60 | -0.45 | 0.326 | N.S |
| 188 | MIR124-3_4 | 98 | -0.45 | 0.326 | N.S |
| 189 | MIR124-3_3 | 83 | -0.42 | 0.336 | N.S |
| 190 | MIR124-1_3 | 60 | -0.37 | 0.356 | N.S |
| 191 | MIR124-3_4 | 132 | -0.91 | 0.364 | N.S |
| 192 | MIR124-3_3 | 31 | -0.32 | 0.376 | N.S |
| 193 | MIR124-3_4 | 129 | -0.87 | 0.39 | N.S |
| 194 | MIR124-3_3 | 143 | -0.86 | 0.391 | N.S |
| 195 | MIR124-3_3 | 138 | -0.25 | 0.401 | N.S |
| 196 | MIR124-3_2 | 103 | -0.24 | 0.407 | N.S |
| 197 | MIR124-3_2 | 148 | -0.82 | 0.413 | N.S |
| 198 | MIR124-3_3 | 200 | -0.20 | 0.422 | N.S |
| 199 | MIR124-3_3 | 50 | -0.18 | 0.429 | N.S |
| 200 | MIR124-3_3 | 115 | -0.78 | 0.439 | N.S |
| 201 | MIR124-3_3 | 108 | 0.75 | 0.454 | N.S |
| 202 | MIR124-3_2 | 93 | -0.06 | 0.475 | N.S |
| 203 | MIR124-3_3 | 25 | -0.70 | 0.485 | N.S |
| 204 | MIR124-1_3 | 49 | -0.67 | 0.507 | N.S |
| 205 | MIR124-1_3 | 103 | 0.04 | 0.517 | N.S |
| 206 | MIR124-1_3 | 117 | 0.11 | 0.542 | N.S |
| 207 | MIR124-3_3 | 58 | -0.57 | 0.573 | N.S |
| 208 | MIR124-3_1 | 31 | 0.40 | 0.656 | N.S |
| 209 | MIR124-3_3 | 161 | 0.43 | 0.665 | N.S |
| 210 | MIR124-3_2 | 95 | 0.45 | 0.674 | N.S |
| 211 | MIR124-3_4 | 70 | -0.42 | 0.679 | N.S |
| 212 | MIR124-3_3 | 149 | 0.55 | 0.71 | N.S |
| 213 | MIR124-1_3 | 98 | 0.62 | 0.731 | N.S |
| 214 | MIR124-3_4 | 87 | 0.35 | 0.731 | N.S |
| 215 | MIR124-3_2 | 162 | 0.74 | 0.77 | N.S |
| 216 | MIR124-1_3 | 127 | -0.28 | 0.784 | N.S |
| 217 | MIR124-3_1 | 37 | -0.20 | 0.846 | N.S |
| 218 | MIR124-1_3 | 200 | 1.07 | 0.858 | N.S |
| 219 | MIR124-3_3 | 78 | 0.13 | 0.899 | N.S |
| 220 | MIR124-3_3 | 69 | -0.12 | 0.903 | N.S |
| 221 | MIR124-3_3 | 94 | 0.11 | 0.916 | N.S |
| 222 | MIR124-3_3 | 119 | -0.03 | 0.979 | N.S |

Table S2. Methylation levels of the three miR-124 precursor genes in 33 MDD patients before and after treatment.

| Target | w0 (mean ± SD) | w8 (mean ± SD) | t or z | p-value | corrected p-value |
| --- | --- | --- | --- | --- | --- |
| MIR124-1_1 | 0.049±0.007 | 0.049±0.005 | 0.87 | 0.809^a^ | N.S |
| MIR124-1_2 | 0.027±0.005 | 0.027±0.004 | 0.64 | 0.740 ^a^ | N.S |
| MIR124-1_3 | 0.084±0.014 | 0.086±0.016 | -0.20 | 0.422 ^a^ | N.S |
| MIR124-1_4 | 0.045±0.007 | 0.045±0.006 | -0.17 | 0.866 | N.S |
| MIR124-2_1 | 0.061±0.016 | 0.063±0.015 | -0.45 | 0.655 | N.S |
| MIR124-2_2 | 0.056±0.008 | 0.057±0.008 | -0.62 | 0.536 | N.S |
| MIR124-2_3 | 0.064±0.013 | 0.063±0.012 | 0.80 | 0.789 ^a^ | N.S |
| MIR124-3_1 | 0.144±0.021 | 0.144±0.018 | 0.09 | 0.931 | N.S |
| MIR124-3_2 | 0.024±0.006 | 0.024±0.005 | 0.91 | 0.819 ^a^ | N.S |
| MIR124-3_3 | 0.044±0.008 | 0.042±0.007 | 0.74 | 0.770 ^a^ | N.S |
| MIR124-3_4 | 0.045±0.007 | 0.045±0.006 | -0.25 | 0.800 | N.S |

^a^ Mann-Whitney test.

Table S3. The statistical results of 222 CpG sites in 33 MDD patients before and after treatment.

|  | **Target** | **Position** | **t or z** | **p-value** |
| --- | --- | --- | --- | --- |
| 1 | MIR124-3_3 | 94 | 3.19 | 0.002 |
| 2 | MIR124-3_3 | 63 | -1.94 | 0.026 |
| 3 | MIR124-3_3 | 161 | 1.78 | 0.079 |
| 4 | MIR124-3_3 | 106 | -1.30 | 0.097 |
| 5 | MIR124-3_4 | 132 | 1.65 | 0.103 |
| 6 | MIR124-3_4 | 62 | -1.22 | 0.112 |
| 7 | MIR124-1_2 | 74 | 1.58 | 0.118 |
| 8 | MIR124-1_3 | 158 | -1.15 | 0.125 |
| 9 | MIR124-3_4 | 215 | -1.51 | 0.137 |
| 10 | MIR124-1_2 | 116 | -1.04 | 0.148 |
| 11 | MIR124-3_2 | 155 | 1.44 | 0.155 |
| 12 | MIR124-1_4 | 161 | 1.41 | 0.165 |
| 13 | MIR124-3_4 | 179 | -1.33 | 0.187 |
| 14 | MIR124-3_4 | 37 | -0.89 | 0.188 |
| 15 | MIR124-2_2 | 108 | -1.32 | 0.193 |
| 16 | MIR124-2_3 | 191 | -0.85 | 0.197 |
| 17 | MIR124-1_4 | 75 | -0.84 | 0.201 |
| 18 | MIR124-2_3 | 203 | 1.26 | 0.213 |
| 19 | MIR124-3_3 | 50 | -0.79 | 0.216 |
| 20 | MIR124-2_1 | 123 | -0.77 | 0.220 |
| 21 | MIR124-1_2 | 40 | -1.23 | 0.224 |
| 22 | MIR124-3_2 | 34 | -1.21 | 0.232 |
| 23 | MIR124-1_2 | 105 | 1.18 | 0.241 |
| 24 | MIR124-1_1 | 152 | -0.69 | 0.245 |
| 25 | MIR124-3_4 | 87 | -0.64 | 0.261 |
| 26 | MIR124-3_2 | 103 | -1.11 | 0.270 |
| 27 | MIR124-3_2 | 182 | -0.61 | 0.272 |
| 28 | MIR124-3_3 | 108 | -1.10 | 0.278 |
| 29 | MIR124-2_2 | 42 | -1.01 | 0.316 |
| 30 | MIR124-3_2 | 162 | 0.98 | 0.332 |
| 31 | MIR124-1_3 | 49 | -0.95 | 0.344 |
| 32 | MIR124-3_3 | 78 | 0.95 | 0.346 |
| 33 | MIR124-3_4 | 46 | -0.94 | 0.350 |
| 34 | MIR124-2_3 | 90 | 0.93 | 0.354 |
| 35 | MIR124-1_4 | 144 | -0.93 | 0.355 |
| 36 | MIR124-3_3 | 31 | -0.37 | 0.356 |
| 37 | MIR124-3_1 | 111 | -0.36 | 0.358 |
| 38 | MIR124-3_2 | 168 | 0.91 | 0.367 |
| 39 | MIR124-3_2 | 64 | 0.91 | 0.367 |
| 40 | MIR124-2_2 | 94 | -0.90 | 0.369 |
| 41 | MIR124-3_3 | 200 | -0.33 | 0.369 |
| 42 | MIR124-2_2 | 99 | -0.29 | 0.386 |
| 43 | MIR124-2_3 | 129 | -0.86 | 0.393 |
| 44 | MIR124-2_3 | 125 | 0.85 | 0.400 |
| 45 | MIR124-1_4 | 96 | -0.83 | 0.412 |
| 46 | MIR124-3_4 | 129 | -0.20 | 0.422 |
| 47 | MIR124-1_1 | 107 | 0.80 | 0.426 |
| 48 | MIR124-3_1 | 95 | -0.80 | 0.427 |
| 49 | MIR124-1_2 | 91 | -0.18 | 0.429 |
| 50 | MIR124-2_3 | 26 | 0.79 | 0.433 |
| 51 | MIR124-3_1 | 62 | -0.16 | 0.437 |
| 52 | MIR124-3_4 | 56 | -0.78 | 0.439 |
| 53 | MIR124-3_3 | 115 | -0.77 | 0.442 |
| 54 | MIR124-2_1 | 117 | -0.76 | 0.452 |
| 55 | MIR124-3_4 | 31 | -0.74 | 0.463 |
| 56 | MIR124-3_2 | 108 | -0.73 | 0.465 |
| 57 | MIR124-3_1 | 100 | -0.06 | 0.475 |
| 58 | MIR124-3_1 | 127 | -0.06 | 0.475 |
| 59 | MIR124-1_2 | 46 | 0.71 | 0.478 |
| 60 | MIR124-1_3 | 117 | -0.03 | 0.489 |
| 61 | MIR124-1_2 | 57 | 0.70 | 0.490 |
| 62 | MIR124-2_2 | 131 | 0.00 | 0.499 |
| 63 | MIR124-3_1 | 87 | 0.00 | 0.499 |
| 64 | MIR124-3_2 | 55 | 0.04 | 0.516 |
| 65 | MIR124-3_3 | 149 | 0.04 | 0.517 |
| 66 | MIR124-3_3 | 25 | 0.63 | 0.529 |
| 67 | MIR124-1_3 | 127 | 0.09 | 0.534 |
| 68 | MIR124-3_4 | 136 | 0.10 | 0.541 |
| 69 | MIR124-1_4 | 148 | -0.61 | 0.541 |
| 70 | MIR124-3_3 | 88 | 0.61 | 0.541 |
| 71 | MIR124-3_1 | 92 | -0.61 | 0.542 |
| 72 | MIR124-1_4 | 169 | -0.61 | 0.542 |
| 73 | MIR124-1_3 | 141 | -0.61 | 0.546 |
| 74 | MIR124-3_3 | 83 | 0.13 | 0.551 |
| 75 | MIR124-3_2 | 119 | -0.59 | 0.556 |
| 76 | MIR124-1_1 | 155 | 0.59 | 0.557 |
| 77 | MIR124-1_2 | 113 | 0.15 | 0.558 |
| 78 | MIR124-3_4 | 74 | 0.15 | 0.558 |
| 79 | MIR124-2_2 | 44 | -0.58 | 0.561 |
| 80 | MIR124-2_2 | 133 | -0.58 | 0.564 |
| 81 | MIR124-3_4 | 51 | 0.58 | 0.564 |
| 82 | MIR124-2_3 | 131 | 0.58 | 0.565 |
| 83 | MIR124-3_2 | 95 | 0.17 | 0.566 |
| 84 | MIR124-3_3 | 69 | -0.58 | 0.567 |
| 85 | MIR124-3_4 | 117 | 0.57 | 0.570 |
| 86 | MIR124-1_3 | 105 | -0.57 | 0.573 |
| 87 | MIR124-3_1 | 106 | 0.57 | 0.573 |
| 88 | MIR124-3_4 | 213 | 0.19 | 0.575 |
| 89 | MIR124-3_2 | 29 | 0.55 | 0.583 |
| 90 | MIR124-1_4 | 176 | 0.21 | 0.584 |
| 91 | MIR124-2_2 | 161 | -0.55 | 0.584 |
| 92 | MIR124-1_1 | 123 | 0.55 | 0.585 |
| 93 | MIR124-1_2 | 44 | 0.23 | 0.592 |
| 94 | MIR124-3_3 | 102 | 0.23 | 0.592 |
| 95 | MIR124-3_4 | 168 | 0.23 | 0.592 |
| 96 | MIR124-3_2 | 140 | -0.54 | 0.593 |
| 97 | MIR124-1_4 | 69 | 0.26 | 0.601 |
| 98 | MIR124-2_1 | 26 | 0.26 | 0.601 |
| 99 | MIR124-3_4 | 240 | -0.51 | 0.610 |
| 100 | MIR124-3_2 | 123 | 0.28 | 0.610 |
| 101 | MIR124-3_1 | 104 | 0.50 | 0.617 |
| 102 | MIR124-1_4 | 61 | 0.30 | 0.619 |
| 103 | MIR124-3_2 | 148 | 0.50 | 0.622 |
| 104 | MIR124-1_1 | 84 | 0.35 | 0.637 |
| 105 | MIR124-1_1 | 144 | 0.35 | 0.637 |
| 106 | MIR124-1_2 | 38 | 0.35 | 0.637 |
| 107 | MIR124-1_2 | 156 | 0.35 | 0.637 |
| 108 | MIR124-1_3 | 103 | 0.36 | 0.640 |
| 109 | MIR124-3_2 | 113 | 0.36 | 0.640 |
| 110 | MIR124-1_4 | 82 | -0.47 | 0.643 |
| 111 | MIR124-3_4 | 70 | 0.46 | 0.646 |
| 112 | MIR124-2_3 | 149 | 0.38 | 0.646 |
| 113 | MIR124-2_3 | 161 | 0.38 | 0.646 |
| 114 | MIR124-3_2 | 67 | 0.38 | 0.646 |
| 115 | MIR124-1_2 | 83 | -0.45 | 0.655 |
| 116 | MIR124-3_2 | 142 | 0.40 | 0.656 |
| 117 | MIR124-1_4 | 42 | 0.44 | 0.660 |
| 118 | MIR124-1_2 | 67 | -0.44 | 0.662 |
| 119 | MIR124-3_1 | 122 | 0.43 | 0.665 |
| 120 | MIR124-3_3 | 138 | 0.43 | 0.667 |
| 121 | MIR124-1_4 | 171 | -0.43 | 0.669 |
| 122 | MIR124-3_2 | 70 | -0.42 | 0.678 |
| 123 | MIR124-3_2 | 40 | 0.48 | 0.683 |
| 124 | MIR124-1_1 | 63 | 0.41 | 0.686 |
| 125 | MIR124-1_3 | 60 | -0.40 | 0.688 |
| 126 | MIR124-1_1 | 87 | 0.40 | 0.691 |
| 127 | MIR124-3_2 | 79 | -0.40 | 0.693 |
| 128 | MIR124-3_1 | 39 | 0.50 | 0.693 |
| 129 | MIR124-1_1 | 148 | 0.40 | 0.694 |
| 130 | MIR124-1_1 | 159 | 0.39 | 0.695 |
| 131 | MIR124-1_4 | 100 | -0.38 | 0.705 |
| 132 | MIR124-1_3 | 200 | -0.38 | 0.705 |
| 133 | MIR124-2_3 | 172 | 0.38 | 0.705 |
| 134 | MIR124-1_1 | 125 | -0.38 | 0.707 |
| 135 | MIR124-3_4 | 83 | -0.37 | 0.714 |
| 136 | MIR124-2_3 | 185 | 0.37 | 0.715 |
| 137 | MIR124-2_1 | 133 | 0.36 | 0.718 |
| 138 | MIR124-2_1 | 87 | -0.36 | 0.720 |
| 139 | MIR124-1_4 | 94 | 0.59 | 0.721 |
| 140 | MIR124-2_3 | 111 | 0.59 | 0.721 |
| 141 | MIR124-3_1 | 37 | 0.59 | 0.721 |
| 142 | MIR124-3_1 | 29 | 0.62 | 0.731 |
| 143 | MIR124-3_1 | 169 | 0.62 | 0.731 |
| 144 | MIR124-2_2 | 146 | -0.33 | 0.744 |
| 145 | MIR124-1_2 | 158 | -0.33 | 0.745 |
| 146 | MIR124-3_1 | 171 | 0.67 | 0.750 |
| 147 | MIR124-2_2 | 101 | 0.31 | 0.759 |
| 148 | MIR124-2_1 | 36 | 0.71 | 0.760 |
| 149 | MIR124-3_3 | 175 | 0.73 | 0.768 |
| 150 | MIR124-3_2 | 57 | 0.74 | 0.770 |
| 151 | MIR124-3_3 | 211 | -0.29 | 0.774 |
| 152 | MIR124-3_2 | 82 | -0.29 | 0.775 |
| 153 | MIR124-1_2 | 87 | -0.28 | 0.783 |
| 154 | MIR124-2_3 | 146 | -0.27 | 0.785 |
| 155 | MIR124-1_1 | 97 | 0.84 | 0.799 |
| 156 | MIR124-1_4 | 92 | -0.25 | 0.802 |
| 157 | MIR124-3_3 | 143 | 0.24 | 0.808 |
| 158 | MIR124-3_2 | 73 | 0.87 | 0.809 |
| 159 | MIR124-3_3 | 85 | 0.24 | 0.812 |
| 160 | MIR124-3_2 | 61 | -0.23 | 0.817 |
| 161 | MIR124-3_2 | 171 | 0.91 | 0.819 |
| 162 | MIR124-3_4 | 139 | 0.91 | 0.819 |
| 163 | MIR124-3_1 | 173 | 0.22 | 0.823 |
| 164 | MIR124-3_1 | 70 | 0.22 | 0.825 |
| 165 | MIR124-2_2 | 34 | -0.21 | 0.832 |
| 166 | MIR124-1_4 | 103 | 0.21 | 0.832 |
| 167 | MIR124-3_1 | 116 | -0.21 | 0.832 |
| 168 | MIR124-3_4 | 106 | -0.21 | 0.833 |
| 169 | MIR124-2_2 | 38 | -0.20 | 0.842 |
| 170 | MIR124-1_2 | 50 | 0.20 | 0.843 |
| 171 | MIR124-3_4 | 53 | 0.20 | 0.846 |
| 172 | MIR124-3_3 | 58 | 0.19 | 0.846 |
| 173 | MIR124-2_1 | 121 | 1.07 | 0.858 |
| 174 | MIR124-2_3 | 35 | 1.07 | 0.858 |
| 175 | MIR124-3_4 | 111 | 1.07 | 0.858 |
| 176 | MIR124-2_2 | 115 | 0.17 | 0.863 |
| 177 | MIR124-3_2 | 138 | -0.17 | 0.864 |
| 178 | MIR124-1_1 | 99 | 1.12 | 0.868 |
| 179 | MIR124-3_1 | 56 | -0.16 | 0.872 |
| 180 | MIR124-3_4 | 243 | -0.16 | 0.874 |
| 181 | MIR124-2_3 | 39 | 1.17 | 0.879 |
| 182 | MIR124-3_4 | 143 | 1.17 | 0.879 |
| 183 | MIR124-3_4 | 234 | 1.17 | 0.879 |
| 184 | MIR124-1_2 | 55 | -0.15 | 0.883 |
| 185 | MIR124-3_3 | 164 | 0.14 | 0.887 |
| 186 | MIR124-3_4 | 76 | 1.22 | 0.889 |
| 187 | MIR124-2_3 | 158 | -0.13 | 0.893 |
| 188 | MIR124-1_4 | 133 | 1.27 | 0.899 |
| 189 | MIR124-3_3 | 168 | 0.12 | 0.905 |
| 190 | MIR124-3_2 | 101 | 0.12 | 0.906 |
| 191 | MIR124-1_2 | 123 | 1.33 | 0.909 |
| 192 | MIR124-3_2 | 76 | 1.33 | 0.909 |
| 193 | MIR124-3_2 | 91 | 0.11 | 0.916 |
| 194 | MIR124-3_4 | 98 | 0.10 | 0.918 |
| 195 | MIR124-2_2 | 51 | -0.10 | 0.923 |
| 196 | MIR124-3_1 | 50 | 0.09 | 0.926 |
| 197 | MIR124-1_1 | 164 | 0.09 | 0.929 |
| 198 | MIR124-2_2 | 74 | 1.47 | 0.929 |
| 199 | MIR124-1_4 | 120 | -0.09 | 0.931 |
| 200 | MIR124-2_1 | 31 | -0.08 | 0.934 |
| 201 | MIR124-1_3 | 98 | 1.54 | 0.939 |
| 202 | MIR124-1_3 | 41 | 1.54 | 0.939 |
| 203 | MIR124-2_3 | 167 | 1.55 | 0.939 |
| 204 | MIR124-1_1 | 131 | -0.07 | 0.945 |
| 205 | MIR124-2_3 | 134 | 0.07 | 0.945 |
| 206 | MIR124-2_1 | 143 | 1.64 | 0.949 |
| 207 | MIR124-3_1 | 77 | 1.64 | 0.949 |
| 208 | MIR124-2_3 | 106 | -0.06 | 0.951 |
| 209 | MIR124-1_2 | 193 | 0.06 | 0.952 |
| 210 | MIR124-1_4 | 153 | 0.06 | 0.952 |
| 211 | MIR124-3_3 | 119 | 0.06 | 0.953 |
| 212 | MIR124-2_2 | 72 | -0.06 | 0.954 |
| 213 | MIR124-1_4 | 139 | 0.05 | 0.958 |
| 214 | MIR124-3_2 | 93 | 0.05 | 0.961 |
| 215 | MIR124-3_3 | 130 | 1.87 | 0.970 |
| 216 | MIR124-2_3 | 94 | 0.03 | 0.975 |
| 217 | MIR124-1_1 | 43 | 2.05 | 0.980 |
| 218 | MIR124-1_1 | 136 | 2.05 | 0.980 |
| 219 | MIR124-3_1 | 120 | 0.02 | 0.987 |
| 220 | MIR124-1_1 | 60 | 2.32 | 0.990 |
| 221 | MIR124-3_3 | 171 | 2.32 | 0.990 |
| 222 | MIR124-3_1 | 31 | Inf | 1.000 |
